# Biological redundancy of endogenous GPCR ligands in the gut and the potential for endogenous functional selectivity

**DOI:** 10.3389/fphar.2014.00262

**Published:** 2014-11-28

**Authors:** Georgina L. Thompson, Meritxell Canals, Daniel P. Poole

**Affiliations:** ^1^Drug Discovery Biology, Monash Institute of Pharmaceutical SciencesParkville, VIC, Australia; ^2^Department of Anatomy and Neuroscience, The University of MelbourneParkville, VIC, Australia

**Keywords:** biased agonism, enteric nervous system, G protein-coupled receptor, somatostatin, somatostatin receptor, opioid, opioid receptor

## Abstract

This review focuses on the existence and function of multiple endogenous agonists of the somatostatin and opioid receptors with an emphasis on their expression in the gastrointestinal tract. These agonists generally arise from the proteolytic cleavage of prepropeptides during peptide maturation or from degradation of peptides by extracellular or intracellular endopeptidases. In other examples, endogenous peptide agonists for the same G protein-coupled receptors can be products of distinct genes but contain high sequence homology. This apparent biological redundancy has recently been challenged by the realization that different ligands may engender distinct receptor conformations linked to different intracellular signaling profiles and, as such the existence of distinct ligands may underlie mechanisms to finely tune physiological responses. We propose that further characterization of signaling pathways activated by these endogenous ligands will provide invaluable insight into the mechanisms governing biased agonism. Moreover, these ligands may prove useful in the design of novel therapeutic tools to target distinct signaling pathways, thereby favoring desirable effects and limiting detrimental on-target effects. Finally we will discuss the limitations of this area of research and we will highlight the difficulties that need to be addressed when examining endogenous bias in tissues and in animals.

## ENDOGENOUS BIASED AGONISM

The last decade has witnessed the experimental confirmation of previous theoretical concepts demonstrating that GPCRs exist in many temporally related micro-conformations (Deupi and Kobilka, 2010). Mechanistically, this inherent plasticity is in line with recent biophysical studies indicating that GPCRs can adopt multiple active states that can be differentially stabilized by chemically distinct classes of ligands (Hofmann et al., 2009; Bokoch et al., 2010). Such plasticity allows GPCRs to mediate a spectrum of acute signaling and longer-term regulatory behaviors that can be activated in a ligand-specific manner. Indeed, it is now established that different agonists do not uniformly activate all cellular signaling pathways linked to a given receptor. Rather, different ligands binding to the same receptor stabilize distinct receptor conformations linked to different signaling pathways and physiological outcomes. This paradigm whereby different ligands, binding to the same GPCR in an identical cellular background, promote distinct receptor conformational states linked to a different functional outcome has been termed *biased agonism* or *functional selectivity*. Therapeutically, biased agonism provides new avenues for the development of drugs that are not only receptor-specific but also ‘pathway-specific.’ As such it has opened the field to the discovery of ligands that selectively activate signaling pathways mediating desired physiological effects whilst minimizing ‘on-target’ side-effects that are elicited by activation of other signaling pathways *via* the same receptor.

To date, most descriptions of biased agonism have focused on the differential effects of synthetic drugs. However, there are several functionally important GPCR families that bind to multiple endogenous agonists [for example chemokine, somatostatin (SST), and opioid receptors (ORs)]. Although this has been traditionally attributed to the redundancy of some biological systems, biased agonism could represent an added layer of control to engender finely tuned physiological responses. Indeed, recent reports have already highlighted the potential for functional selectivity across the chemokine receptor family (Rajagopal et al., 2013; Zweemer et al., 2014).

In this focused review we provide an overview of the existing literature regarding two of these GPCR families with multiple endogenous peptide ligands, opioids and SST, in the context of the gastrointestinal tract (GIT). The opioid system is a prototypical example of potential biological redundancy, and it also represents one of the first examples where functional selectivity of synthetic drugs has been reported in the context of gut physiology. On the other hand, SST receptors are therapeutic targets in treating GI disease (e.g., diarrhea, bleeding varices, neuroendocrine tumors) and SSTs and related peptides also represent a well-characterized system where multiple endogenous ligands of the same receptor exist within the GIT (Zhao et al., 2013). Importantly, these two receptor systems also reveal different mechanisms that can explain distinct physiological outcomes derived from activation of the same receptor by different ligands.

## THE SOMATOSTATIN SYSTEM OF THE GUT

There are five members of the SST receptor family (SSTR_1-5_) and their distribution in the GIT has been recently reviewed (Van Op den Bosch et al., 2009).

Somatostatin, originally known as somatotropin release-inhibiting factor (SRIF), was first identified and characterized as a cyclic tetradecapeptide (Brazeau et al., 1973). It was predicted that SST-14 was a product of a larger peptide precursor and that other forms with potential biological activity were likely to exist. Indeed, it is now known that SST arises from maturation of preprosomatostatin (PPSST), and that all PPSST derivatives originate from the *SST* gene. The removal of a 24 amino acid signal sequence forms prosomatostatin (PSST), which is further C-terminally cleaved to form the biologically active peptides SST-14, SST-25, and SST-28 (Bohlen et al., 1980; Esch et al., 1980; Brazeau et al., 1981). SST-28, the longest peptide, was identified and characterized as an N-terminally extended variant of SST-14 (Pradayrol et al., 1978, 1980; Bohlen et al., 1980) and biological conversion of SST-28 to SST-14 was later confirmed (Zingg and Patel, 1983). Other cleavage products arising from PSST processing include PSST(1–32; Schmidt et al., 1985) and PSST(1–64; Bersani et al., 1989), for which little information regarding function and expression is available.

N-terminal cleavage of PSST also occurs, but the resulting peptides do not contain the SST-14 sequence and are therefore not considered to be SSTs (Benoit et al., 1990). These include SST-28(1–12) and antrin, which contains amino acids 1–10 of PSST [PPSST(25–34)]. Antrin, first identified in the gastric antrum (Benoit et al., 1987), is present in all SST-producing tissues. However, a functional role for this peptide has yet to be ascribed. Most recently, a bioinformatics approach predicted the existence of a novel 13mer PPSST cleavage product [PPSST(31–43)], which was subsequently confirmed by immunoaffinity purification and called neuronostatin (Samson et al., 2008). Neuronostatin is encoded by PSST and is highly conserved across vertebrates. Unlike SST and cortistatin (CST, see below), neuronostatin is not cyclic and is amidated at the C-terminus.

Biological activity of SST variants is conferred through a common Phe-Trp-Lys-Thr (FWKT) motif within the C-terminus (amino acids 7–10; Patel and Srikant, 1997). This sequence is also present in non-SST peptides that share a high-degree of sequence homology with SST. These include CST and thrittene. CST and SST are encoded by distinct genes, and genetic deletion of SST has no effect on the expression of CST. CST is a derivative of the 112 amino acid preproCST (PPCST) precursor (de Lecea et al., 1996), which is converted to proCST by signal peptide cleavage, resulting in the formation of hCST17 and hCST29 (Puebla et al., 1999). CST shares 11 amino acids in common with SST-14 including residues required for interaction with SSTRs and two key cysteines that enable formation of the cyclic peptide structure (Francis et al., 1990). Although they share sequence homology, structure, and affinity for SSTRs, there are clear differences in the ability of CST and SST-14 to stimulate SSTR2 endocytosis and signaling (Liu et al., 2005). Notably, CST is significantly less effective at inhibiting cAMP production and promoting SSTR2 endocytosis. Furthermore, CST does not exclusively interact with SST receptors and can also activate the MrgX2 and GHS-R1a receptors. Whether there are CST variants or a CST-specific GPCR is unknown. Another endogenous peptide that shares extensive sequence homology with SST is thrittene [SST28(1–13)]. As with CST, thrittene is not derived from PSST and is a product of a distinct gene, as supported by the presence of thrittene-like immunoreactivity in PSST deficient mice (Ensinck et al., 2003). Moreover, thrittene and SST are expressed by distinct cell populations and their release is triggered in response to different stimuli (Ensinck et al., 2002). With the exception of these initial studies nothing is known of the functional role of thrittene, nor if thrittene plays an analogous or discrete role to that of SST. A summary of SST and SST-like peptides is presented in **Table 1**.

**Table 1:** Endogenous somatostatin (SST) peptide sequences (*sequence not confirmed).

Precursor	Peptide	Sequence
Prosomatostatin (PSST)	SST-28	Ser-Ala-Asn-Ser-Asn-Pro-Ala-Met-Ala-Pro-Arg-Glu-Arg-Lys-Ala-Gly-Cys-Lys-Asn-Phe-Phe-Trp-Lys-Thr-Phe-Thr-Ser-Cys
	SST-14	Ala-Gly-Cys-Lys-Asn-Phe-Phe-Trp-Lys-Thr-Phe-Thr-Ser-Cys-OH
	Neuronostatin (PPSST(31-43))	Leu-Arg-Gln-Phe-Leu-Gln-Lys-Ser-Leu-Ala-Ala-Ala-Ala-NH2
	Antrin (SST-25-34)	Ala-Pro-Ser-Asp-Pro-Arg-Leu-Arg-Gln-Phe-OH
	SST-25	Ser-Asn-Pro-Ala-Met-Ala-Pro-Arg-Glu-Arg-Lys-Ala-Gly-Cys-Lys-Asn-Phe-Phe-Trp-Lys-Thr-Phe-Thr-Ser-Cys
	SST-28(1–14)	Ser-Ala-Asn-Ser-Asn-Pro-Ala-Met-Ala-Pro-Arg-Glu-Arg-Lys
	SST-28(1–12)	Ser-Ala-Asn-Ser-Asn-Pro-Ala-Met-Ala-Pro-Arg-Glu
	PPST 1–64	Ala-Pro-Ser-Asp-Pro-Arg-Leu-Arg-Gln-Phe-Leu-Gln-Lys-Ser-Leu-Ala-Ala-Ala-Ala-Gly-Lys-Gln-Glu-Leu-Ala-Lys-Tyr-Phe-Leu-Ala-Glu-Leu-Leu-Ser-Glu-Pro-Asn-Gln-Thr-Glu-Asn-Asp-Ala-Leu-Glu-Pro-Glu-Asp-Leu-Ser-Gln-Ala-Ala-Glu-Gln-Asp-Glu-Met-Arg-Leu-Glu-Leu-Gln-Arg
	PSST 1–32	Ala-Pro-Ser-Asp-Pro-Arg-Leu-Arg-Gln-Phe-Leu-Gln-Lys-Ser-Leu-Ala-Ala-Ala-Ala-Gly-Lys-Gln-Glu-Leu-Ala-Lys-Tyr-Phe-Leu-Ala-Glu-Leu
Preprocortistatin	Cortistatin-14 (rat)	Pro-Cys-Lys-Asn-Phe-Phe-Trp-Lys-Thr-Phe-Ser-Ser-Cys-Lys
	Cortistatin-17 (human)	Asp-Arg-Met-Pro-Cys-Arg-Asn-Phe-Phe-Trp-Lys-Thr-Phe-Ser-Ser-Cys-Lys
	Cortistatin-29	H-Glu-Gly-Ala-Pro-Pro-Gln-Gln-Ser-Ala-Arg-Arg-Asp-Arg-Met-Pro-Cys-Arg-Asn-Phe-Phe-Trp-Lys-Thr-Phe-Ser-Ser-Cys-Lys-OH
Unknown	Thrittene (SST28(1–13))	Ser-Ala-Asn-Ser-Asn-Pro-Ala-Met-Ala-Pro-Arg-Glu-Arg*

### DISTRIBUTION OF ENDOGENOUS SSTR LIGANDS IN THE GI TRACT

The GIT is the major source of SST and SST is a regulator of many digestive functions. SSTRs are an important therapeutic target in the treatment of digestive disease. In addition to its established role as a neurotransmitter, SST also acts in a hormonal and paracrine manner to regulate gut function (Low, 2004; Van Op den Bosch et al., 2009).

Somatostatin is expressed by D-cells of the stomach and plays a well-defined role in the control of acid secretion. SST negatively regulates gastrin release from antral G cells and histamine release from enterochromaffin-like cells, and acts directly on parietal cells leading to an SSTR2-dependent inhibition of acid release (Walsh, 1988; Lloyd et al., 1997; Low, 2004). SST-14 within the intestinal wall is mainly expressed by enteric neurons and potentially by extrinsic primary spinal afferents (Traub et al., 1999), although this is still debated (Keast and De Groat, 1992). SST-14 is also produced by macrophages during infection or inflammation as part of an immunoregulatory circuit with SSTR2 (Weinstock and Elliott, 2000). SST-28- distribution appears to be more restricted and is primarily expressed by enteroendocrine D-cells (Ravazzola et al., 1983; Baskin and Ensinck, 1984), consistent with the predominant release of SST-28 from the mucosa (Baldissera et al., 1985).

Myenteric SST-immunoreactivity is localized to a subclass of descending inhibitory interneuron, where it is co-expressed with choline acetyltransferase (Portbury et al., 1995; Song et al., 1997). Physiologically, SST is involved in the migrating myoelectric complex in the jejunum (Abdu et al., 2002) and propagating contractions of the colon (Grider, 2003). These actions are mediated through the SSTR2 receptor, which is expressed by NOS-positive inhibitory motor neurons or descending interneurons (Allen et al., 2002). SST is also an inhibitor of gastric emptying and of gall bladder contractility. SST is expressed by submucosal cholinergic secretomotor/ non-vasodilator neurons (Mongardi Fantaguzzi et al., 2009) and hyperpolarizes submucosal neurons (Shen and Surprenant, 1993) probably *via* SSTR1 and SSTR2 (Foong et al., 2010). In the human intestine SST is expressed by putative intrinsic primary afferent neurons of the submucosal plexus (Beyer et al., 2013).

There is limited information regarding the distribution of ‘non-SST’ peptides in the gut. Relatively high mRNA expression for CST has been detected through the human GIT (Dalm et al., 2004). However, it should be noted that with the exception of pancreatic delta islet cells (Papotti et al., 2003) and potentially activated inflammatory cells (Gonzalez-Rey et al., 2006), the distribution of CST within the gut remains unknown. Thrittene-like immunoreactivity has been detected in enteroendocrine cells and enteric neurons and this distribution is distinct to that for SST-14 and SST-28 (Ensinck et al., 2002). This is supported by the differential release of thrittene and SST in response to feeding (Ensinck et al., 2003). Antrin expression was originally believed to be restricted to gastric D-cells, where it is localized to SST-28(1–12) containing secretory granules (Ravazzola et al., 1989; Benoit et al., 1990). However, this was contradicted by Rabbani and Patel (1990), who demonstrated comparable expression of antrin in the jejunal mucosa and pancreas by radioimmunoassay and HPLC.

### EVIDENCE FOR DIFFERENCES IN FUNCTION

At present there is little evidence for significant differences in the effects of endogenous SSTs on GI function, although this may reflect the limited endpoints that have been assayed. Exposure of enteric neurons to SST results in activation of inwardly rectifying K^+^ currents and to hyperpolarization, leading to inhibition of contractile and secretory activity (Van Op den Bosch et al., 2009). Direct electrophysiological recordings demonstrate no apparent difference in the acute effects of SST-14 and SST-28 on submucosal neurons, with exposure to either agonist leading to hyperpolarization and to rapid desensitization of responses (Shen and Surprenant, 1993). Similarly, there was no significant difference in the SST-14, SST-25, and SST-28 mediated inhibition of contractile activity. These agonists cross-desensitized responses to each other, but not to acetylcholine, suggesting actions at the same receptor (McIntosh et al., 1986). However, there is evidence for differences in the *in vivo* effects of SST-14 and SST-28 on both the stomach and intestine. For example, studies examining the direct effects of SSTR activation on gut function showed that SST-14 is significantly more potent at inhibiting gastric acid secretion than SST-28, despite the longer plasma half-life of SST-28 (Hirst et al., 1982; Seal et al., 1982). Zhao et al. (2013) recently demonstrated that although SST-14 and SST-28 both stimulated endocytosis of SSTR2A in myenteric neurons, there were clear differences in receptor recycling. The apparent retention of SSTR2A following treatment of neurons with SST-28 was attributed to the greater resistance of this peptide to degradation by the endosomal endopeptidase endothelin-converting enzyme 1 (ECE-1). This study did not determine the consequences of this retention or prolonged endosomal SSTR2A signaling on gut function. Moreover, the possible biological activity of SST cleavage products resulting from ECE-1 activity was not examined. Intermediate products of both SST-14 (SST-1–10) and SST-28 (SST-1–24) retained the Phe-Trp-Lys-Thr motif at the extreme N-terminus and may represent novel SSTR agonists produced locally within endosomes. However, absence of a key N-terminal Cys residue suggests that these peptides lack the cyclopeptide structure characteristic of SSTs.

The existence of endogenous ligand bias has been examined at the SSTR2A. Comparison of the responses of SST-14, SST-28 and cortistatin has not showed any evidence of functional selectivity at this receptor. However, potential ligand bias has been suggested for the small molecule ligands that bind SSTR2A, albeit the quantification of this bias is lacking (Nunn et al., 2004; Liu et al., 2008; Cescato et al., 2010). More recently, we have shown that SST-14 and SST-28 show distinct profiles of receptor trafficking upon internalization (Zhao et al., 2013). After incubation with SST-14, SSTR2A recycled to the plasma membrane, which required the activity of the endosomal peptidase ECE-1, and an intact Golgi. In contrast, SSTR2A activated by SST-28, octreotide, lanreotide, or vapreotide was retained within the Golgi and did not recycle. Although ECE-1 rapidly degraded SST-14, SST-28 was resistant to degradation, and ECE-1 did not degrade the synthetic SST analogs. Thus, although no apparent bias was observed at the level of receptor signaling events, SST-14 and SST-28 differ in the trafficking of the receptor upon internalization. The differential regulation of SSTR2A may explain the different physiological effects of endogenous agonists and could account for the long-lasting therapeutic actions and side effects of clinically used agonists.

## THE OPIOID SYSTEM IN THE GUT

Opioids and opiates are agonists of the mu, delta and kappa ORs (MOPr, DOPr, and KOPr). The nociceptin receptor (NOPr) was the last ORs to be cloned and is grouped with the ORs based on their high degree of sequence homology and its low level binding of opioids. The pharmacology and function of ORs has been reviewed extensively and will not be covered in detail in this review (Waldhoer et al., 2004). All receptors are expressed by enteric neurons and other cell types in the GIT and are major regulators of gut function (Wood and Galligan, 2004; Galligan and Akbarali, 2014)

The endogenous ligands for ORs are a large family of at least 20 different small peptides. The endogenous peptides have been detected throughout the central and peripheral nervous system as well as in other tissues, with similar distribution to the ORs. They are involved in numerous physiological processes including nociception, reward processing, and GIT motility and secretion. The distribution and physiological effects of endogenous opioids in the GIT have been the most extensively studied. However, identifying regions where endogenous opioids are expressed and released under normal physiological conditions has been challenging due to the high susceptibility of the peptides to degradation. Additionally, most studies have used antibody-based methods that may not reliably distinguish between different opioid peptides due to their high structural similarity, or HPLC-based methods which provide no detail of the specific cell types that express these peptides. Further complications arise due to interspecific differences and region-dependent variations in expression along the GIT. Nonetheless, most of the endogenous opioids are present in the GIT, and in some cases the distribution and release from discrete regions of the GIT has been thoroughly characterized.

There are three major classes of endogenous opioids (enkephalins, dynorphins, and endorphins), which are synthesized by proteolytic cleavage of precursor proteins; pro-enkephalin, pro-dynorphin, and pro-opiomelanocortin (POMC), respectively. The peptides range from 5 to 30 amino acids in length, and share a common N-terminal tetrapeptide sequence Tyr-Gly-Gly-Phe, with either a Leu or Met in the fifth position. These peptides have varying affinities for all three ORs, but none are highly selective for one receptor subtype (Mansour et al., 1995; Janecka et al., 2004). There are also two additional putative endogenous peptides; endomorphin-1, and endomorphin-2, which are structurally unrelated to the typical opioid peptides and are most selective and potent for MOPr (Zadina et al., 1997). The gene or genes encoding the precursor proteins of endomorphins are unknown (Terskiy et al., 2007), although a *de novo* synthesis mechanism has been proposed as an alternative source (Ronai et al., 2009). The presence of endomorphins in the GIT has not been reported and will not be discussed further in this review.

### DISTRIBUTION OF ENDOGENOUS OPIOID RECEPTOR LIGANDS IN THE GI TRACT

Screening of the longitudinal muscle-myenteric plexus of the guinea pig ileum by HPLC identified expression of enkephalins (enk: Leu-enk, Met-enk, Met-enk-Arg-Gly-Leu, Met-enk-Arg-Phe, Metorphamide, and BAM-18) and dynorphins [α-neoendorphin, β-neoendorphin, dynorphin A(1–8), and dynorphin B]. No detectable beta endorphin was present in these preparations (Corbett et al., 1988).

#### Enkephalins

**FIGURE 1:**
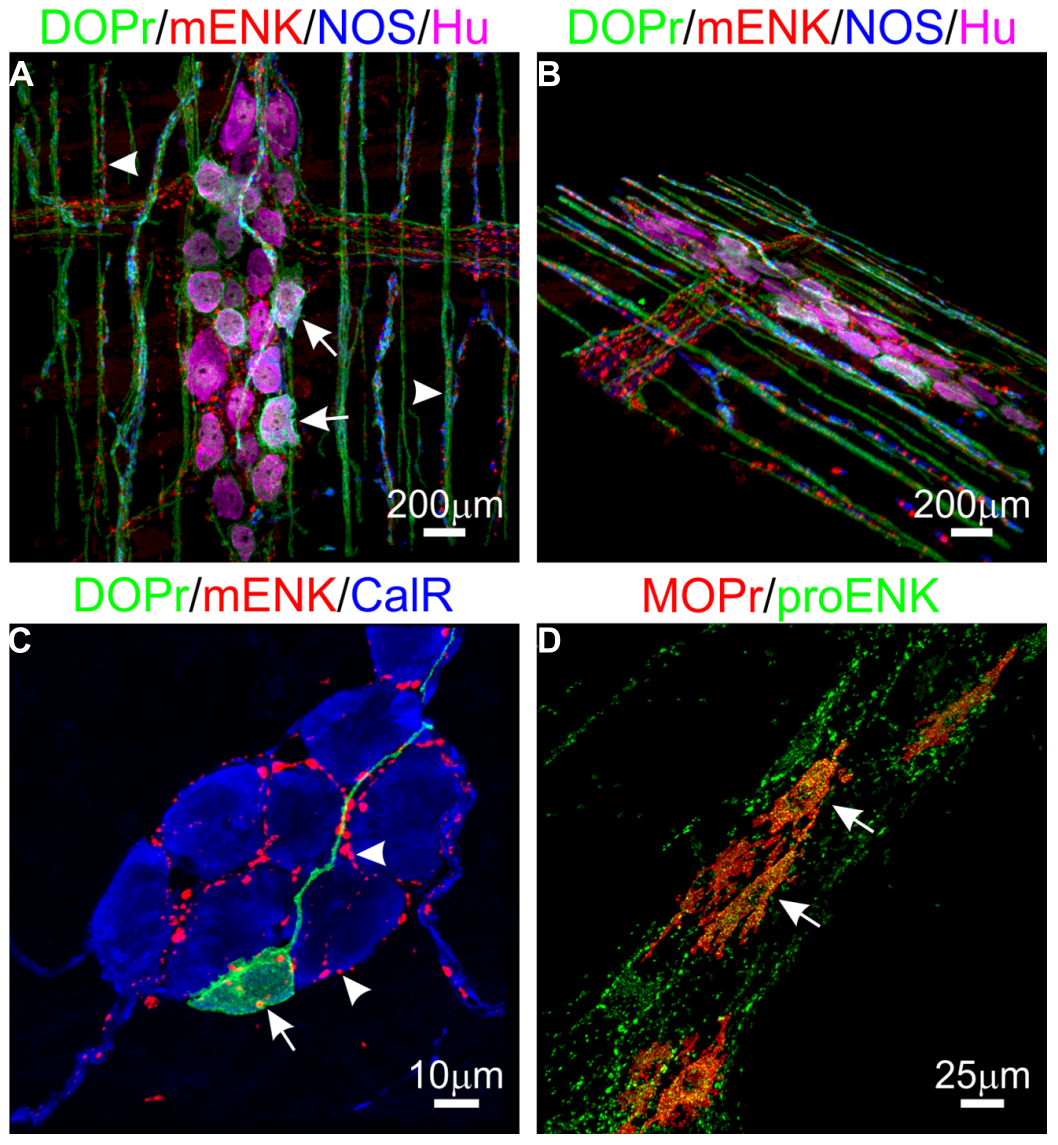
**Expression of opioid receptors (ORs) and enkephalin in the enteric nervous system. (A,B)** Distribution of the delta opioid receptor (DOPr, green), met-enkephalin (mENK, red), nitric oxide synthase (NOS, blue), and the pan-neuronal marker HuC/D (Hu, magenta) in the myenteric plexus (arrows) and circular muscle nerve fibers (arrowheads) of the mouse distal colon. **(C)** Example of a DOPr-positive submucosal neuron (arrow) and association with mENK-immunoreactive nerve varicosities (arrowheads) in the mouse distal colon. **(D)** Overlap between immunoreactivities for the Mu opioid receptor (MOPr, red) and proenkephalin (proENK, green) in myenteric neurons of the guinea pig ileum. Images have been modified using Imaris 7.4.2 software (Bitplane). Scale bars are as indicated.

The enkephalins have been the most widely studied opioid peptides in the GIT. Pro-enkephalin contains four copies of Met-enk and one each of Leu-enk, Met-enk-Arg-Phe, and Met-enk-Arg-Gly-Leu, and several additional opioid peptides may be formed by partial processing of the precursor protein (see **Table 2**; Noda et al., 1982). Expression of at least four enkephalin peptides (Leu-enk, Met-enk, Met-enk-Arg-Phe, and Met-enk-Arg-Gly-Leu) in the GIT has been confirmed (Hughes et al., 1977; Linnoila et al., 1978; Tang et al., 1982; Giraud et al., 1984). Immunohistochemical studies demonstrate expression throughout the human GIT, with highest levels detected in the *muscularis externa* (Polak et al., 1977; Ferri et al., 1986, 1988). A similar expression pattern has been observed in rodents (Keast et al., 1985). Enkephalin-derived peptides are mainly found in the cell bodies of myenteric neurons and in nerve fibers within the myenteric plexus and circular muscle (Elde et al., 1976; Jessen et al., 1980; Schultzberg et al., 1980; Furness et al., 1983). There is evidence that immunoreactivities for Leu-enk and Met-enk are expressed by distinct neuronal populations within the enteric nervous system (Linnoila et al., 1978; Larsson et al., 1979; Larsson and Stengaard-Pendersen, 1982). The morphology and distribution of Enk-containing myenteric neurons has been examined in detail. Approximately 23% of myenteric neurons express Enk-immunoreactivity (Furness et al., 1983). These are morphologically Dogiel Type I inhibitory or excitatory motor neurons and are also immunoreactive for ChAT and/ or substance P (Furness et al., 1983; Bornstein et al., 1984; Costa et al., 1985; Pfannkuche et al., 1998). Leu-enk-positive myenteric neurons of the human intestine have been described morphologically as ‘stubby neurons’ and are proposed to represent motor neurons or ascending interneurons (Brehmer et al., 2005). Examples of OR and enkephalin labeling in the intestine are presented in **Figure 1**.

**Table 2:** Endogenous opioid peptide sequences.

Precursor	Peptide	Sequence
Pro-Enkephalin	Leu-enkephalin	Tyr-Gly-Gly-Phe-Leu
	Met-enkephalin	Tyr-Gly-Gly-Phe-Met
	Met-enkephalin-Arg-Phe	Tyr-Gly-Gly-Phe-Met-Arg-Phe
	Met-enkephalin-Arg-Gly-Leu	Tyr-Gly-Gly-Phe-Met-Arg-Gly-Leu
	Metorphamide	Tyr-Gly-Gly-Phe-Met-Arg-Arg-Val
	BAM 12	Tyr-Gly-Gly-Phe-Met-Arg-Arg-Val-Gly-Arg-Pro-Glu
	BAM 18	Tyr-Gly-Gly-Phe-Met-Arg-Arg-Val-Gly-Arg-Pro-Glu-Trp-Trp-Met-Asp-Tyr-Gln
	BAM 22	Tyr-Gly-Gly-Phe-Met-Arg-Arg-Val-Gly-Arg-Pro-Glu-Trp-Trp-Met-Asp-Tyr-Gln-Lys-Arg-Tyr-Gly
	Peptide E	Tyr-Gly-Gly-Phe-Met-Arg-Arg-Val-Gly-Arg-Pro-Glu-Trp-Trp-Met-Asp-Tyr-Gln-Lys-Arg-Tyr-Gly-Gly-Phe-Leu
	Peptide F	Tyr-Gly-Gly-Phe-Met-Lys-Lys-Met-Asp-Glu-Leu-Tyr-Pro-Leu-Glu-Val-Glu-Glu-Glu-Ala-Asn-Gly-Gly-Glu-Val-Leu-Gly-Lys-Arg-Tyr-Gly-Gly-Phe-Met
Pro-Dynorphin	Dynorphin A	Tyr-Gly-Gly-Phe-Leu-Arg-Arg-Ile-Arg-Pro-Lys-Leu-Lys-Trp-Asp-Asn-Gln
	Dynorphin B	Tyr-Gly-Gly-Phe-Leu-Arg-Arg-Gln-Phe-Lys-Val-Val-Thr
	Big Dynorphin (Dyn A/B 1-32)	Tyr-Gly-Gly-Phe-Leu-Arg-Arg-Ile-Arg-Pro-Lys-Leu-Lys-Trp-Asp-Asn-Gln-Lys-Arg-Tyr-Gly-Gly-Phe-Leu-Arg-Arg-Gln-Phe-Lys-Val-Val-Thr
	Dynorphin A 1–13	Tyr-Gly-Gly-Phe-Leu-Arg-Arg-Ile-Arg-Pro-Lys-Leu-Lys
	Dynorphin A (1–8)	Tyr-Gly-Gly-Phe-Leu-Arg-Arg-Ile
	Dynorphin A (1–6)	Tyr-Gly-Gly-Phe-Leu-Arg
	Leumorphin	Tyr-Gly-Gly-Phe-Leu-Arg-Arg-Gln-Phe-Lys-Val-Val-Thr-Arg-Ser-Gln-Glu-Asp-Pro-Asn-Ala-Tyr-Tyr-Glu-Glu-Leu-Phe-Asp-Val
	α-neoendorphin	Tyr-Gly-Gly-Phe-Leu-Arg-Lys-Tyr-Pro-Lys
	β-neoendorphin	Tyr-Gly-Gly-Phe-Leu-Arg-Lys-Tyr-Pro
Pro-Opiomelanocortin	α-endorphin	Tyr-Gly-Gly-Phe-Met-Thr-Ser-Glu-Lys-Ser-Gln-Thr-Pro-Val-Thr-Leu
	β-endorphin	Tyr-Gly-Gly-Phe-Met-Thr-Ser-Glu-Lys-Ser-Gln-Thr-Pro-Val-Thr-Leu-Phe-Lys-Asn-Ile-Ile-Lys-Asn-Ala-Tyr-Lys-Lys-Gly-Glu
Unknown	Endomorphin 1	Tyr-Pro-Trp-Phe
	Endomorphin 2	Tyr-Pro-Phe-Phe

There are a small number of neurons that express enkephalin-immunoreactivity in the submucosal plexus and fibers in the mucosa (Furness et al., 1985; Keast et al., 1985; Pfannkuche et al., 1998), and in enteroendocrine cells (Mimoda et al., 1998). However, it is possible that the enkephalin detected in these regions is due to detection of dynorphins or dynorphin derived Leu-enk which is highly expressed in these regions as discussed later in this review. Expression of other enkephalin derivatives including Met-enk-Arg-Phe (Bu’Lock et al., 1983) and Met-enk-Arg-Gly-Leu (Wang and Lindberg, 1986) by enteric neurons has also been demonstrated. Other sites where preproenkephalin and its derivatives are expressed include enteroendocrine cells (Bu’Lock et al., 1983; Nihei and Iwanaga, 1985; Kokrashvili et al., 2009), extrinsic afferents (Steele and Costa, 1990) and immune cells including CD4+ T cells (Boue et al., 2014).

#### Dynorphins

There is good evidence that opioid peptides derived from pro-dynorphin (dynorphins), are present in the GIT. Pre-pro-dynorphin mRNA is expressed in the myenteric and mucosal layers to varying levels throughout the GIT (Yuferov et al., 1998). Prodynorphin contains three opioid peptides, dynorphin A, dynorphin B, and α-neoendorphin, which can all be further processed to shorter opioid peptides including Leu-Enk (see **Table 2**; Horikawa et al., 1983). Dynorphins have been detected in the GIT of various species, including the full length Dyn A (1–17), Dyn A (1–13), Dyn A (1–8), Dyn B, and α-neoendorphin (Vincent et al., 1984; Wolter, 1986; Steele et al., 1989; Murphy and Turner, 1990; Spampinato et al., 1992). Dynorphins are present in all layers of the gut wall throughout the entire human GIT, although information regarding cellular sites of expression is lacking (Spampinato et al., 1988). Immunohistochemistry studies performed mainly in guinea pigs indicate that dynorphins are widely expressed by submucosal and myenteric neurons (Vincent et al., 1984; Wolter, 1986; Steele and Costa, 1990). Dynorphins are co-expressed with enkephalins in a subpopulation of Dogiel type I myenteric neurons (Costa et al., 1985; Furness et al., 1985; Steele and Costa, 1990). It is possible that this may reflect conversion of dynorphin to Leu-enk in these neurons rather than co-expression of pro-enkephalin. There are also reports of dynorphin expression by enterochromaffin cells (Cetin, 1988).

#### Endorphins

The endorphins are formed from the precursor peptide POMC, which also contains several other non-opioid peptide hormones (Eipper and Mains, 1978). POMC contains only one opioid peptide, β-endorphin, which can be cleaved to form α-endorphin. Although β-endorphin has been detected in the GIT (Orwoll and Kendall, 1980; DeBold et al., 1988), the localization of endorphin expression still remains uncertain. There is some evidence of β-endorphin expression, and of other POMC peptides, by myenteric neurons, nerve fibers within the circular muscle and enteroendocrine cells (Schultzberg et al., 1980; Leander et al., 1984; Wolter, 1985b; Kokrashvili et al., 2009; Miller and Hirning, 2010). Another major source of β-endorphin in the gut are immune cells, particularly those associated with inflammatory bowel disease or irritable bowel syndrome (Verma-Gandhu et al., 2006; Hughes et al., 2013). It should be noted that the distribution of β-endorphin in the GIT is controversial, as the specificity of the antisera used in many of these studies has been questioned (Sundler et al., 1981). Hence whilst there is certainly β-endorphin present in the GIT, the question of its origin remains unresolved.

Other OR agonists are also produced endogenously in the GIT. These include morphine and codeine-like compounds (Schulz et al., 1977; Laux-Biehlmann et al., 2013) and the pre-dermorphin derivatives dermorphin and dermenkephalin (Mor et al., 1989, 1990).

Even though the distribution of the different classes of endogenous opioids in the GIT has been fairly well established, there is very little known about individual levels of the different peptides within each class. The expression of proteases that synthesize and degrade endogenous opioids may have varying levels of expression in different cell types, which would result in different production and degradation rates. As such, the mixture of opioid peptides derived from the same precursor will be variable in different cell populations. Differential proteolytic processing of pro-enkephalin and pro-dynorphin peptides occurs in various regions of the brain and other tissues, leading to variations in the relative proportions of peptides derived from the same precursors (Cone et al., 1983; Zamir et al., 1984; Yakovleva et al., 2006). Differential processing of precursors may also occur in the different cell populations within the GIT. In rat duodenum, specific antisera against Dyn A (1–17) and Dyn A (1–8) stain two distinct populations of neurons, one which contains both peptides and one with only Dyn A (1–8), indicating that Dyn A (1–8) may be synthesized *via* distinct proteases or at varying rates in distinct neuronal populations (Wolter, 1985a).

### FUNCTION OF ENDOGENOUS OPIOID RECEPTOR LIGANDS IN THE GI TRACT

Endogenous opioids play an important regulatory role in normal gut physiology, primarily through activation of ORs on enteric neurons (Wood and Galligan, 2004). When applied exogenously, the physiological effects of endogenous opioids are the same as the effects of other opioids, they hyperpolarize enteric neurons leading to inhibition of GIT motility and secretion and ultimately cause constipation (Miller and Hirning, 2010). On the other hand, the effects of endogenous peptides when released intrinsically under normal physiological conditions are unclear. Release of enkephalin- and dynorphin-derived peptides has been detected in intestinal tissue preparations during peristalsis or after electrical stimulation. These include Leu-enk, Met-enk, Met-enk-Arg-Phe, Met-enk-Arg-Gly-Leu, metorphamide (Schulz et al., 1977; Corbett et al., 1991), α-neoendorphin (Majeed et al., 1987) and Dyn A (Kromer et al., 1981; Donnerer et al., 1984). In addition, studies using opioid antagonists, mainly naloxone, have shown that inhibition of opioid activity increases non-propagating intestinal motility (Sanger and Tuladhar, 2004). Altogether, this shows that endogenous opioids play a subtle but important role in control of GIT motility by suppressing activity. There is also evidence that the endogenous peptides either contribute to, or protect against, the development of pathophysiological conditions. Levels of endogenous opioids in the GIT have been shown to increase under pathological conditions, including inflammatory bowel disease, and not only inhibit gastrointestinal motility, but also provide visceral antinociception. β-endorphin levels have been shown to increase in a model of chronic inflammatory bowel disease in mice, suppressing inflammation-associated hyperexcitability of colonic primary spinal afferents (Hughes et al., 2013; Valdez-Morales et al., 2013). In addition, T Lymphocytes can release β-endorphin and induce expression of β-endorphin in the myenteric plexus in mice with immunodeficiency-related visceral hyperalgesia (Verma-Gandhu et al., 2006, 2007). Surgical intervention has also been shown to increase dynorphin expression in the dorsal root ganglia of mice (Romero et al., 2012), and stimulate release of opioid peptides from enteric neurons after abdominal surgery in guinea pigs (Patierno et al., 2005). This may contribute in part to post-operative ileus, although sympathetic pathways are likely to play a more significant role. A greater understanding of the involvement of endogenous opioids in GIT pathophysiology is important as the opioid system is not only a potential target for treatment, but the enhanced production and release of endogenous opioids may also alter the effectiveness of opioid-based therapeutics.

Although the global physiological effects of endogenous opioids in the GIT have been widely studied, the role of individual peptides in the control of normal GIT functions or pathophysiological conditions in discrete regions is still not clear. There are specific distributions of endogenous opioids throughout the GIT. However, since all endogenous opioids can activate all ORs, the specific ORs through which endogenous opioids exert their actions or the specific signaling mechanisms behind these functions is unknown. The physiological significance of such diversity and structural organization of opioid peptides suggests that individual endogenous peptides may serve distinct physiological roles. The diversity in physiological effects can in part be achieved by activation of the different ORs. However, as there are far more endogenous opioids than there are receptors and little receptor selectivity, it is probable that the diversity in endogenous opioids exists to fine tune OR-mediated effects through biased agonism.

### BIASED AGONISM AT THE OPIOID RECEPTORS

Opioid receptors are prototypical GPCRs where biased agonism displayed by synthetic and exogenous ligands has been widely explored. Indeed, this reflects the extensive knowledge of opioid physiology and the desire to generate opioid-based analgesics devoid of limiting side effects such as respiratory depression or constipation.

In addition to the ideal separation of therapeutic and clinically limiting side effects, two key observations in the actions of morphine at MOPr have sparked the search for biased agonists at this receptor. First, morphine is relatively poor at inducing MOPr internalization, in spite of its efficacy in mediating G-protein activation, and second, morphine-induced respiratory depression and constipation were attenuated in a β-arrestin knock-out mouse, while analgesia was enhanced. Altogether these reports have sparked the search for potentially different signaling mechanisms that mediate the diverse physiological actions of ORs. Similarly, reports of biased agonism by exogenous ligands have also been described for the other OR subtypes, DOPr (Charfi et al., 2014), and KOPr (Melief et al., 2010). However, the potential for endogenous bias at the OR family has not received much attention. This is despite the fact that, as highlighted above, there is significant biological redundancy in the opioid system. In a systematic approach to evaluate biased agonism at the mu-OR, McPherson et al. (2010; Rivero et al., 2012) examined the signaling bias of a wide range of ligands including endogenous opioid peptides and synthetic opioids. In these and subsequent studies, endomorphin-2 as well as endomorphin-1 showed statistically significant bias toward β-arrestin2 recruitment and away from G protein activation. However, as neither the gene nor the precursor protein of endomorphin1 and two has yet been identified, their classification as endogenous opioids is still a matter of debate.

Opioid receptors have also been reported to form homo- and hetero-dimers. Importantly, it has been suggested that these dimers may indeed form a new signaling entity where the intracellular signaling resulting from the activation of heterodimers may be different from that elicited by the individual protomers or homodimers (Waldhoer et al., 2005; Rozenfeld and Devi, 2007; Gomes et al., 2013). Moreover, some of these dimers have been demonstrated to exist *in vivo* (Massotte, 2014). Although such mechanisms of engendering distinct intracellular signals would not fall into the definition of biased agonism, it is another paradigm to take into account in the context of the differential actions of endogenous opioid peptides.

## IDENTIFICATION AND QUANTIFICATION OF BIASED AGONISM: CHALLENGES AND LIMITATIONS

Although biased agonism offers the potential of safer and more effective therapeutics, there are still significant limitations for its detection, quantification and, importantly, its translation into differential physiological responses.

### QUANTIFICATION OF BIASED AGONISM

Analytical tools for the detection and quantification of biased agonism are necessary in order to effectively inform future drug development efforts aimed in this direction. The majority of studies to date on biased agonism have used largely qualitative observations, such as reversals in agonist potency orders or maximal agonist effects between different pathways. However, such approaches are not optimal. The potency of a ligand is determined by both its affinity for the receptor state coupled to that particular pathway as well as its intrinsic efficacy for generating a response in that pathway. In contrast, the maximal effect of a ligand at saturating concentrations is only determined by intrinsic efficacy. In addition, contributors to system bias, signal amplification, and receptor expression need to be taken into account as they have markedly different effects on potencies and efficacies of differently efficacious ligands. Therefore, the observed response of an agonist at a given pathway is not only the result of unique ligand-induced receptor conformations, rather it is affected by “system bias,” which reflects the differing coupling efficiencies of the receptor to a given signaling pathway, and by “observation bias,” which results from differing assay sensitivity and conditions (Kenakin et al., 2012; Kenakin and Christopoulos, 2013a). It is the bias imposed by the ligand on the receptor that is the only source of bias that allows the signaling bias profiles of ligands in different cell types to be compared. It is therefore important to quantify signaling bias in such a way that it excludes system and observation bias, in order to reveal the unique signaling profile that is induced by the different ligands.

Several analytical approaches have been described to quantify biased agonism (reviewed by Kenakin and Christopoulos, 2013b). The relative transduction ratio (Kenakin et al., 2012) is one of the most robust and widely applicable methods for bias quantification. This method applies the operational model of agonism first derived by Black and Leff (1983) to concentration-response curves and estimates a “transduction coefficient” which is comprised of the functional equilibrium dissociation constant (a measure of the affinity for the receptor coupled to a particular effector protein or signaling pathway) and the intrinsic efficacy of the agonist in activating a particular signaling response and receptor density. This coefficient is thus an overall measure of the relative ‘power’ of an agonist to induce a response. In order to eliminate the effects of system and observation bias, normalization to a reference agonist is required. Finally, these normalized transduction coefficients can be compared across two signaling pathways for a given agonist to obtain the “relative transduction ratio” as measures of agonist bias. It is, however, important to highlight that key factors need to be considered [reference ligand, cellular content and pluridimensionality of efficacy, (Thompson et al., 2014)] which influence the identification and quantification of biased agonism and that need to be taken into account when interpreting information obtained from studying biased signaling *in vitro*.

### EXAMINATION OF ENDOGENOUS BIAS IN A PHYSIOLOGICAL SETTING

Potentially insurmountable difficulties may prevent the examination of endogenous ligand bias in tissues and *in vivo*. First and foremost, multiple agonists for the same receptor exist, and these may be coexpressed (e.g., enkephalins), precluding differential release protocols. Selective stimulation of release may be possible in cases where agonists are expressed by distinct cells or neuronal subtypes (e.g., enteric neurons *vs*. enteroendocrine cells). Peptides may differ with respect to their susceptibility to degradation, complicating interpretation of studies of duration or magnitude of effects. Furthermore, these peptides may vary in their relative affinities to receptors of interest. The endpoints that are measured are often indirect and result from activation of complex reflex pathways involving a number of transmitters. For example, suppression of electrically evoked intestinal contractions, such as occurs in response to OR agonists (Wood and Galligan, 2004) may not reveal subtle agonist-dependent differences. Most of the current descriptions of biased agonism rely on direct measurements from cells (e.g., pERK1/2, cAMP accumulation, β-arrestin-recruitment), which are difficult to assay in enteric neurons. Moreover, the effects of exogenous agonist application may not reflect what occurs physiologically, as location of receptors and ligands may mean that such interactions never occur.

Other factors to consider when translating data derived from heterologous cell lines to enteric neurons, tissues, or *in vivo* studies include not only species, but also regional differences, and the relative expression of key regulatory proteins in the cellular environment examined. These factors are most apparent in the case of the ORs. The distribution of ORs in the gut differs between species. For example, there is limited evidence for functional DOPr expression in the guinea pig ileum (Johnson et al., 1987), whereas there is prominent DOPr expression in the mouse ileum (Poole et al., 2011). There may also be differences in the regional distribution of ORs with respect to both the relative numbers of positive neurons and in the neuronal types that express these receptors, as we have previously demonstrated for the DOPr (Poole et al., 2011). Interestingly, this does not appear to be the case for MOPr expression in the guinea pig ileum and colon where similar neuronal populations express the receptor (Poole et al., unpublished). It is worth noting that these differences in distribution are unlikely to have an effect in the detection of bias, as measurements are likely to be performed in the same tissue preparation. However, species and regional differences in OR expression will affect the potential for heterodimerization of ORs, which may influence the pharmacological profiles of any responses to agonists (Rozenfeld and Devi, 2007). Perhaps of greater importance is the relative expression of key modulatory proteins including β-arrestins and GRKs, which influence OR signaling in enteric neurons. This is highlighted by a number of recent studies using knockout mice. β-arrestin 2 deficient mice exhibit reduced constipatory effects of morphine and loperamide based on assays of fecal output and colonic transit (Raehal et al., 2005). Similarly, GRK6^-/-^ mice also display significantly diminished opiate-induced inhibition of colonic transit relative to wildtype mice (Raehal et al., 2009). Deletion of either β-arrestin 2 or GRK6 did not affect morphine-induced inhibition of small intestinal transit, suggesting region-dependent regulation of neuronal MOR. β-arrestins are also integral to the development of opiate tolerance in the intestine, with deletion of β-arrestin 2 promoting acute morphine tolerance in the colon (Maguma et al., 2012; Akbarali et al., 2014). These studies highlight that OR regulation and physiological function can differ markedly between regions of the GIT and the difficulty in translating data obtained from model cell systems to the physiological setting.

In summary, we have provided an overview of the expression and distribution of endogenous ligands for two major therapeutically relevant classes of GPCRs in the GIT. We have provided evidence for functional selectivity of these ligands and have discussed potential issues related to translation of cell line-derived data to the organ and whole animal levels. Therapeutically, the targeting of selective release of endogenous peptides is probably not a realistic goal. However, understanding the fundamental basis for ligand bias and determining whether differences in the expression and release of endogenous ligands underlie the development and maintenance of disease may be more promising avenues to address and to provide mechanistic insight for the development of safer therapies.

## Conflict of Interest Statement

The authors declare that the research was conducted in the absence of any commercial or financial relationships that could be construed as a potential conflict of interest.
